# Global Council on Brain Health: Consensus and Recommendations for Promoting Brain Health Through Music Engagement

**DOI:** 10.1093/geroni/igaa057.2836

**Published:** 2020-12-16

**Authors:** Jacobo Mintzer

**Affiliations:** Roper St. Francis Healthcare, Charleston, South Carolina, United States

## Abstract

This presentation will summarize the points of consensus reached by the experts and describes the major points of discussion that led to their recommendations for men and women age 50 and older. The GCBH considered questions surrounding what happens in the brain when listening to music as well as creating music. Topics such as music therapy and the clinical dimensions of introducing music into caring for patients will be addressed. Alongside the consensus points, recommendations and practical tips will be presented. Moreover, we will also identify gaps in our knowledge that currently exist in the evidence base surrounding music and brain health.

